# Surgical Management of Bilateral Venous Malformation (Cavernous Hemangiomas) of the Maxillary Sinus

**DOI:** 10.1155/2020/8606103

**Published:** 2020-02-10

**Authors:** Takashi Anzai, Shin Ito, Atsushi Yamashita, Takuma Ide, Shori Tajima, Hiroko Okada, Fumihiko Matsumoto, Katsuhisa Ikeda

**Affiliations:** ^1^Department of Otorhinolaryngology, Juntendo University, Faculty of Medicine, Tokyo, Japan; ^2^Department of Human Pathology, Juntendo University, Faculty of Medicine, Tokyo, Japan

## Abstract

According to International Society for the Study of Vascular Anomalies classification 2018, “hemangioma” should be classified as either vascular tumor or vascular malformation (VM). So-called “cavernous hemangioma” is categorized as VM. VM rarely involves the mucous membranes of the sinonasal cavity and typically arises unilaterally from the sinonasal cavity. Bilateral VM of the maxillary sinus is extremely rare. To the best of our knowledge, there is no previous report of bilateral VM of the maxillary sinus. Here, we describe the surgical treatment of bilateral cavernous hemangiomas of the maxillary sinus. These tumors were successfully resected by endoscopic modified medial maxillectomy (EMMM) after embolization. Endoscopic sinus surgery, particularly EMMM, produces access to the bilateral maxillary sinus and can prevent several complications.

## 1. Introduction

According to International Society for the Study of Vascular Anomalies classification 2018, “hemangioma” should be classified as either vascular tumor or vascular malformation (VM) [1]. So-called “cavernous hemangioma” is categorized as VM. It rarely involves the mucous membranes of the sinonasal cavity and typically arises unilaterally in the sinonasal cavity. Typically, VM is associated with the lateral wall of the nasal cavity or the inferior turbinates and is rarely associated with the maxillary sinus [2, 3]. To the best of our knowledge, there is no previous report of bilateral VM of the maxillary sinus. The mainstay of treatment for VM is complete surgical excision. Here, we describe the surgical treatment of bilateral VM of the maxillary sinus. These tumors were successfully resected by endoscopic modified medial maxillectomy (EMMM) after embolization.

## 2. Case Report

**Figure 1:**
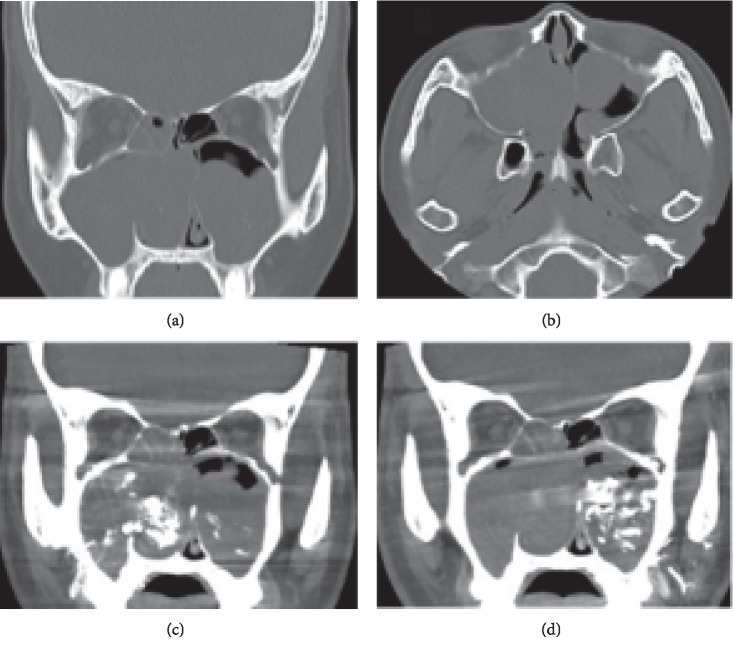
Noncontrast computed tomography and computed tomography angiography images. Preoperative (a) coronal and (b) axial noncontrast computed tomography shows a soft tissue mass filled the bilateral nasal cavities and maxillary sinuses without bony destruction. Computed tomography angiography shows that (c) the main feeder of right-side tumor was greater palatine artery and (d) the other side was infraorbital artery.

**Figure 2:**
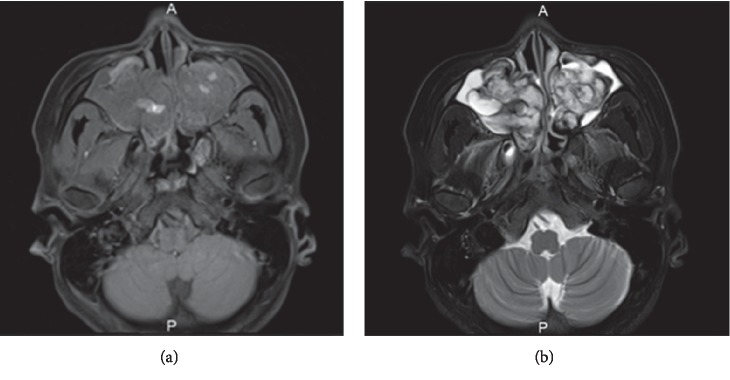
Magnetic resonance imaging (MRI). The mass as heterogeneous signal intensity arises from the bilateral maxillary sinus and extend to the nasal cavity on (a) T1-weighted image and (b) T2-weighted image.

**Figure 3:**
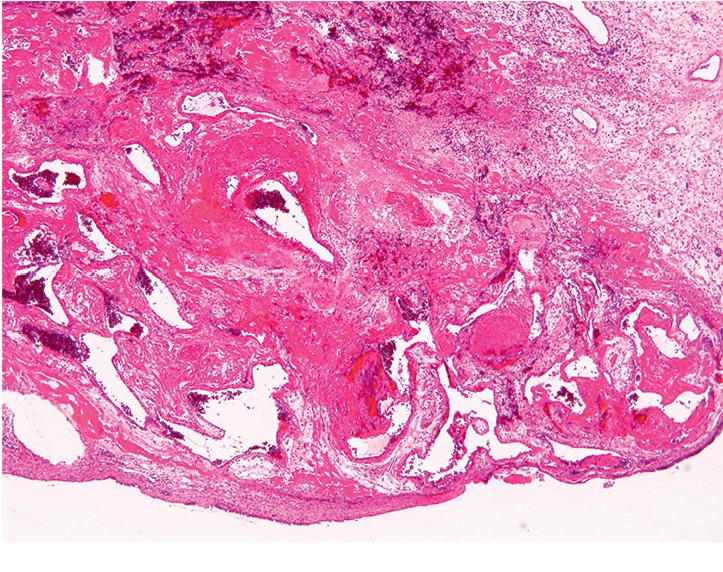
Histopathological analysis of specimen using hematoxylin and eosin (H&E) stain ×40. The H&E sections show dilated vascular spaces that are lined by endothelial cells without significant atypia.

**Figure 4:**
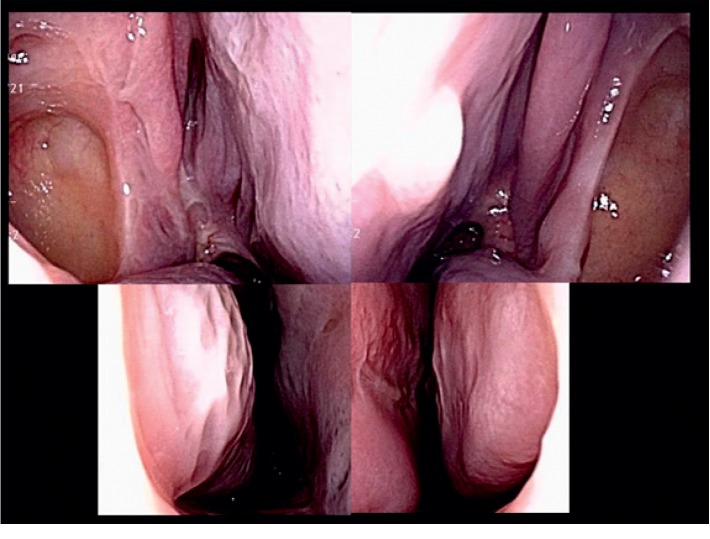
Postoperative endoscopic findings. The endoscopic findings showed a normal appearance.

**Figure 5:**
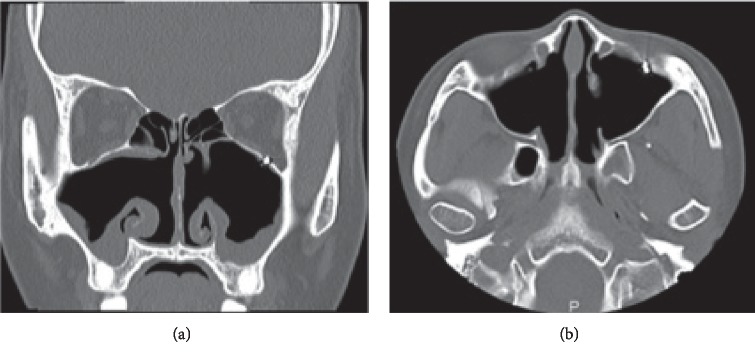
Computed tomography (CT) image (postoperation). Postoperative (a) coronal and (b) axial CT scans show the both sides of nasolacrimal duct and inferior turbinate were preserved. Also, the coil in the left infraorbital artery was seen in infraorbital canal.

A 28-year-old woman presented to the Department of Otolaryngology, Juntendo University Hospital, with frequent epistaxis and nasal obstruction. Ten years ago, she had received surgical treatment in another hospital, the details of which were unknown to us. Anterior rhinoscopy revealed a reddish mass that filled both nasal cavities. Computed tomography (CT) showed a soft tissue mass that filled the bilateral nasal cavities and maxillary sinuses without bony destruction (Figures 1(a) and 1(b)). Computed tomography angiography showed that the main feeder of right-side tumor was right greater palatine artery (Figure 1(c)) and that of left-side tumor was left infraorbital artery (Figure 1(d)). Magnetic resonance imaging showed the mass as having heterogeneous signal intensity and arising from the bilateral maxillary sinus and extending into the nasal cavity on T1- and T2-weighted images (Figure 2). These findings were indicative of a VM. To avoid severe hemorrhage during operation, the right greater palatine artery and left orbital artery were selectively embolized using metallic coils before the operation. Endoscopic sinus surgery (ESS) was then performed under general anesthesia. Because the tumor fully occupies the maxillary sinus on both sides, EMMM was performed on both sides. A mucosal incision was made from the anterior part of the lateral nasal wall. The mucosa was elevated from the lateral nasal wall. The lateral nasal wall was removed, and the nasolacrimal ducts were exposed with a diamond burr. The inferior turbinates and nasolacrimal ducts were completely preserved. The inferior turbinates and nasolacrimal ducts were displaced medially. Enlarged resection of the lateral wall allowed complete removal of the tumors in both maxillary sinuses. These tumors contained partially necrotic tissue due to preoperative embolization. The operating time was 113 min, and the intraoperative blood loss was 80 mL. Permanent histopathologic examination of the tumor showed large blood-filled spaces lined with flattened endothelium, and the tumor was found to be a VM, so-called cavernous hemangioma (Figure 3). The postoperative course was uneventful. The endoscopic view revealed preservation of both the inferior turbinates (Figure 4). Postoperative CT showed that both sides of the nasolacrimal ducts and inferior turbinates were preserved (Figure 5). Nasal breathing was found to be satisfactory. No nasolacrimal duct obstruction was observed. The patient showed no evidence of disease recurrence at 12 months postoperatively.

## 3. Discussion

Clinically, VM can be sporadic or inherited. It was reported that the mutant TIE2, a member of the receptor tyrosine kinase subfamily, with a kinase domain, is a trigger and pathogenic core [4]. Bilateral VM of the maxillary sinus is extremely rare. Our case has no family history of VM. Thus, somatic mutation might cause bilateral VM of the maxillary sinus.

The mainstay of treatment for VMs is complete surgical excision. Maxillary sinus VMs are usually resected with surgical techniques that provide wide excision, e.g., Caldwell–Luc approach and lateral rhinotomy. Recently, a purely transnasal endoscopic approach has been employed [5]. Endoscopic medial maxillectomy is a safe and effective approach for the treatment of maxillary sinus diseases [6, 7]. Additionally, endoscopic modified medial maxillectomy (EMMM) enables preservation of the inferior turbinates and nasolacrimal ducts [8, 9].

Recently, EMMM has been validated as an effective treatment for maxillary benign tumors. If the tumors occupy bilateral maxillary sinuses, it is also a good indication [10]. We applied EMMM to preserve both the inferior turbinates and nasolacrimal ducts. VM rising bilaterally from the maxillary sinus can be completely removed with good visualization. Our patient did not experience empty nose syndrome or excessive tearing postoperatively.

Preoperative transarterial embolization may be useful to decrease intraoperative blood loss and improve visualization [11]. On the other hand, it has potential of severe complication. Kovalerchik et al. reported successful endoscopic resection of angiofibroma in nasal cavity without preoperative embolization [12]. The risks and benefits of preoperative embolization have to be carefully considered. It is difficult to establish common indications for preoperative arterial embolization. A criteria in our institution to perform preoperative embolization is the large size case which is the origin of the tumor cannot be detected clearly via a nasal endoscope and/or the case which deep seated in the nasal cavity or paranasal sinus. In this middle to large size of recurrent VM case, the origin of the tumor cannot be detected via a nasal endoscope. If the tumor is originated from anterior wall or lateral wall of maxillary sinus, it may be difficult to coagulate under a microscope. So, we performed preoperative embolization.

In conclusion, ESS including EMMM may be successfully used to achieve the complete removal of VM arising bilaterally from the maxillary sinus.
